# Fish Consumption and Mercury Exposure among Louisiana Recreational Anglers

**DOI:** 10.1289/ehp.1002609

**Published:** 2010-10-27

**Authors:** Rebecca A. Lincoln, James P. Shine, Edward J. Chesney, Donna J. Vorhees, Philippe Grandjean, David B. Senn

**Affiliations:** 1 Department of Environmental Health, Harvard School of Public Health, Boston, Massachusetts, USA; 2 Louisiana Universities Marine Consortium, Chauvin, Louisiana, USA; 3 Department of Environmental Health, Boston University School of Public Health, Boston, Massachusetts, USA; 4 Institute of Biogeochemistry and Pollutant Dynamics, Swiss Federal Institute of Technology, Zürich, Switzerland

**Keywords:** angler, fish, hair, Louisiana, mercury, methylmercury

## Abstract

**Background:**

Methylmercury (MeHg) exposure assessments among average fish consumers in the United States may underestimate exposures among U.S. subpopulations with high intakes of regionally specific fish.

**Objectives:**

We examined relationships among fish consumption, estimated mercury (Hg) intake, and measured Hg exposure within one such potentially highly exposed group, recreational anglers in the state of Louisiana, USA.

**Methods:**

We surveyed 534 anglers in 2006 using interviews at boat launches and fishing tournaments combined with an Internet-based survey method. Hair samples from 402 of these anglers were collected and analyzed for total Hg. Questionnaires provided information on species-specific fish consumption during the 3 months before the survey.

**Results:**

Anglers’ median hair Hg concentration was 0.81 μg/g (*n* = 398; range, 0.02–10.7 μg/g); 40% of participants had levels >1 μg/g, which approximately corresponds to the U.S. Environmental Protection Agency’s reference dose. Fish consumption and Hg intake were significantly positively associated with hair Hg. Participants reported consuming nearly 80 different fish types, many of which are specific to the region. Unlike the general U.S. population, which acquires most of its Hg from commercial seafood sources, approximately 64% of participants’ fish meals and 74% of their estimated Hg intake came from recreationally caught seafood.

**Conclusions:**

Study participants had relatively elevated hair Hg concentrations and reported consumption of a wide variety of fish, particularly locally caught fish. This group represents a highly exposed subpopulation with an exposure profile that differs from fish consumers in other regions of the United States, suggesting a need for more regionally specific exposure estimates and public health advisories.

Ample evidence has shown that human exposure to methylmercury (MeHg) can cause adverse health effects [Mergler et al. 2007; National Research Council (NRC) 2000]. The vast majority of human exposure to MeHg occurs through consumption of fish and shellfish (NRC 2000), and long-running studies in the Seychelles (Davidson et al. 2008) and Faroe Islands (Grandjean 2007) have documented neurocognitive deficits in children exposed *in utero*. Among adults, moderate levels of exposure to MeHg have been linked to decreased neuropsychological function (Yokoo et al. 2003) and increased risk of cardiovascular disease (Choi et al. 2009; Virtanen et al. 2005), although there remains a lack of consensus on this latter association given the presence of beneficial nutrients in seafood (e.g., Mozaffarian and Rimm 2006; Stern 2007).

Studies over the past decade have generated a substantial body of data on typical MeHg exposure within the general U.S. population (e.g., McDowell et al. 2004) and have evaluated the associated population-level risks (Mahaffey et al. 2004). They have shown that typical U.S. residents consume moderate amounts of fish [16.9 g/day; U.S. Environmental Protection Agency (EPA) 2002] and are exposed to low to moderate levels of MeHg (0.19 μg/g in hair; McDowell et al. 2004). The types and sources of fish consumed by the general U.S. population are also of interest, because levels of MeHg in fish vary substantially by species [Food and Drug Administration (FDA) 2006] and by region of origin (e.g., Burger and Gochfeld 2006). In a recent study, Sunderland (2007) used commercial fish market data to show that typical U.S. consumers derive most of their MeHg exposure from a narrow range of fish and shellfish types (e.g., tuna, swordfish, and pollock), most of which are imported, not locally caught or farmed.

However, levels and sources of fish consumption and MeHg exposure in the United States vary substantially, and limited data are available to characterize potential highly exposed subpopulations. Several recent studies identified U.S. subpopulations with high fish consumption and MeHg exposure due to cultural preferences and practices (e.g., McKelvey et al. 2007), “high-end” diets (consisting of expensive predator fish; Hightower and Moore 2003), and regional access to fresh seafood (through coastal residence) (Mahaffey et al. 2009). One group in particular that merits further study is U.S. recreational anglers, whose MeHg exposure is still not well documented. The limited work that has been carried out both in the United States (Gobeille et al. 2006; Knobeloch et al. 2007) and internationally (e.g., Al-Majed and Preston 2000; Kosatsky et al. 2000) suggests that recreational anglers are likely to be highly exposed because of high consumption of wild-caught, regionally specific fish, which may exhibit particularly high MeHg concentrations.

Coastal Louisiana is home to a large, avid recreational fishing community as well as a highly productive fishery in the Gulf of Mexico (Chesney et al. 2000). In 2006, approximately 780,000 Louisiana residents, almost 20% of the state’s population, purchased a recreational fishing license (U.S. Fish and Wildlife Service 2009), and both anglers and nonanglers consume fish at comparatively high rates (Dellenbarger et al. 1993). We hypothesized that recreational Louisiana anglers have elevated MeHg exposures relative to the general U.S. population through frequent consumption of both recreationally caught and commercially sourced fish. We explored this hypothesis in a cross-sectional study of fish consumption and MeHg exposure among recreational anglers in coastal Louisiana, using hair samples and dietary data from more than 600 participants. The goals of the study were to measure the anglers’ exposure to MeHg, assess the types and amounts of fish consumed and their contribution to anglers’ MeHg exposure, and determine the dietary information necessary to predict this exposure.

## Materials and Methods

### Study population

We recruited recreational anglers for participation in the study from May through November 2006 in two ways: “in- person” participants (*n* = 225) were recruited by study personnel at boat launches and fishing tournaments in coastal Louisiana; “web-based” participants (*n* = 438) took an Internet-based version of the same survey, which was promoted through a variety of media outlets targeted to Louisiana anglers (web sites, newspapers, and radio shows). Criteria for inclusion in the study were age ≥ 18 years, Louisiana residence, and at least one recreational fishing trip in the past 3 months. Both in-person and web-based participants provided informed consent before beginning the survey.

Of the 663 anglers recruited through both methods, 129 were excluded because they did not meet the inclusion criteria detailed above (*n* = 66) or because they did not complete the interview (*n* = 63; primarily web-based participants). After exclusions, the sample included 534 anglers (in-person *n* = 196; web-based *n* = 338).

### Survey design and administration

The survey instrument [see Supplemental Material, Appendix A (doi:10.1289/ehp.1002609)] was developed by combining fish consumption questions, modified from a semiquantitative food frequency questionnaire (FFQ) used in the Nurses’ Health Study (Hu et al. 2002), with an additional set of questions to characterize anglers’ fishing practices, fish consumption during the 3 months before the survey, sources of consumed fish (recreational vs. commercial), and demographic information. In-person surveys were administered by interviewers trained to query anglers in a standard, consistent manner. The web-based survey was identical to the in-person survey in content but was formatted so that it could be self-administered.

The research protocol, survey instrument, and consent procedures were reviewed and approved by the Harvard School of Public Health (HSPH) Human Subjects Committee before recruitment.

### Hair sample collection and analysis

At the completion of the survey, all participants were asked to submit a hair sample for mercury (Hg) analysis. Overall, 402 of 534 eligible anglers (75%) provided a sample: 181 in-person (response rate = 92%) and 221 web-based participants (response rate = 65%).

The hair samples of the in-person participants were collected by the interviewer. A bundle of hairs approximately 3 mm in diameter was cut from the occipital region of the head using stainless steel scissors and tied with unwaxed dental floss to mark the proximal end.

After completing the online survey, web-based participants were mailed a kit containing detailed instructions and materials for cutting a hair sample. All instructions and materials were identical to those used for in-person hair sample collection. Web-based participants were asked to mail their sample back to HSPH within 30 days using a preaddressed, stamped envelope.

Two centimeters of the proximal end of each sample were analyzed for total Hg by thermal decomposition, amalgamation, and atomic absorption spectrophotometry [EPA method 7473 (U.S. EPA 2007); Milestone Direct Mercury Analyzer; Milestone Inc., Shelton, CT, USA). MeHg makes up the majority of total Hg in hair (80–90%; Institute of Medicine 2007), and total Hg in hair is a reliable biomarker of MeHg intake from fish consumption (Grandjean et al. 2002). Precision and accuracy of this method were confirmed through repeated analysis of standards of known concentration; additional details on analysis, quality control, and detection limits are provided in the Supplemental Material [see Section 1 (doi:10.1289/ehp.1002609)]. Four hair samples were excluded because of insufficient sample size, leaving a final *n* of 398 (in-person *n* = 177; web-based *n* = 221) for all analyses involving hair Hg concentration. Within 6 months of the sample collection, each participant was mailed a letter that contained the results of their analysis, along with guidance on how to interpret that result and further information on MeHg in fish.

### Fish consumption and Hg dose

The survey’s recall period (3 months) was chosen to approximately coincide with the exposure period represented by the hair biomarker: 1–3 months before the survey [see Supplemental Material, Section 2 (doi:10.1289/ehp.1002609)]. We assessed anglers’ fish consumption over this time period using two approaches. In the “overall” approach, anglers were asked to indicate the frequency that best fit their actual finfish and shellfish consumption from the following choices: never, once a month or less, once a week, three times a week, once a day, more than once a day. In the analysis we combined the two lowest categories as well as the two highest categories because of low numbers in those groups. In the “species-specific” approach, anglers were asked to indicate the frequency that best fit their actual consumption of each of 28 common recreational and commercial fish, from the following choices: never, once in the past 3 months, once a month, once a week, three times a week, once or more a day. Participants were also prompted to report consumption of any fish types not included in the list. Each participant’s consumption frequency for each species was converted to a number of meals per day, based on a recall period of 91 days (3 months), and summed to give participants’ total species-specific fish consumption.

To quantify anglers’ Hg intake, fish Hg concentration data were gathered from a variety of sources. These ranged from regionally specific monitoring databases (e.g., Louisiana Department of Environmental Quality 2010; U.S. EPA 2000) to federal databases maintained by the U.S. EPA (2003) and FDA (2006). Additional details on the databases and fish Hg concentration values used in the Hg dose calculations are presented in the Supplemental Material [see Section 3 and Table 1 (doi:10.1289/ehp.1002609)]. In general, only data on total Hg in fish were available, so these values were used to quantify the total Hg doses of the participants. Speciation studies have shown that 90–100% of total Hg in most finfish is MeHg (Bloom 1992). Thus, in most cases total Hg intake via fish consumption can be used as a reasonable proxy for MeHg exposure, but to maintain the distinction between what is measured and what is relevant to health, we refer throughout to Hg intake or dose in contrast to MeHg exposure.

Two Hg dose metrics were calculated to explore how well questionnaire data predicted Hg levels in anglers’ hair. The first Hg dose metric, referred to as the “species-specific” dose, was calculated as follows:


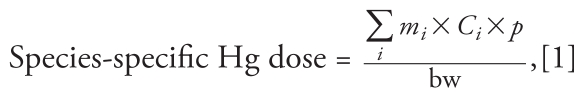


(micrograms Hg per kilogram body weight per day), where for each fish type *i*, *m* represents number of meals per day, *C* represents Hg concentration (micrograms per gram), *p* represents a standard portion size (129 g; U.S. EPA 1997), and bw represents self-reported body weight (kilograms).

The second Hg dose metric, referred to as the “scaled” Hg dose, was constructed by calculating each participant’s average species-specific Hg dose per fish meal (representing a measure of the Hg richness of the fish diet) and then multiplying this by the overall fish consumption frequency. This scales the species-specific fish consumption down by the level of overall fish consumption while still retaining species-specific Hg information, and attempts to account for potential overreporting in the species-specific fish consumption variable. It is calculated as follows:


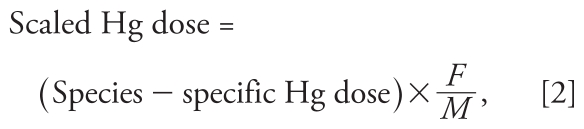


(micrograms Hg per kilogram body weight per day), where *F* represents overall fish consumption in meals/day and *M* represents species-specific fish consumption in meals/day.

### Statistical methods

We first explored the data using scatter plots and descriptive statistics to identify departures from normal distributions. The distribution of hair Hg concentrations was positively skewed, and a log-transformation of the variable was used in subsequent analyses. Variables describing fish consumption and Hg dose were also non-normally distributed, and nonparametric tests were used where necessary.

The relationships among anglers’ fish consumption, Hg doses, and hair Hg concentrations (considered here to be the most accurate indicator of MeHg exposure) were explored using multivariable linear regression. Three separate regression models were created, relating overall fish consumption, species-specific Hg dose, and scaled Hg dose to hair Hg. Age, sex, education, race/ethinicity, body mass index (BMI; calculated from self-reported height and weight), and survey type were included in each model as potentially important covariates. Calculated Hg dose metrics, age, and BMI were treated as continuous variables. Overall fish consumption, sex, race/ethinicity, and education were treated as categorical variables.

Residual plots for each model were examined to ensure that standard assumptions of linearity, normality, and homoskedasticity were met. Studentized residuals and Cook’s *D* values were calculated to identify potential outliers and influential points in each model. Several influential points were identified, and the original surveys and laboratory output data for these points were reexamined and found to be error free. We compared results of our original linear regressions with robust regressions, which reduce the effect of extreme values by weighting points in proportion to their leverage (Hampel et al. 1986). Our findings did not change when robust regression was used; thus, the results presented are those from the original linear regressions.

All analyses were performed using R version 2.6.2 (R Development Core Team 2008). The level of statistical significance was set at α = 0.05.

## Results

### Participant characteristics and hair Hg levels

Participants were predominantly male (89%) and white/Caucasian (96%), with a mean age of 45 years (range, 19–84 years) and a mean BMI of 28.3. Twenty-four percent attained a high school education or less, 24% completed some college, 31% received a college degree, and 21% pursued postgraduate education.

Hg levels in anglers’ hair ranged from 0.02 to 10.7 μg/g, with a median of 0.81 μg/g and a mean of 1.1 μg/g (Table 1). Among all participants, 38% had a hair Hg concentration > 1 μg/g, the level that approximately corresponds to the EPA’s reference dose of 0.1 μg/kg/day (Rice et al. 2003), and 13% had a hair Hg concentration > 2 μg/g, a level that has been associated with adverse cardiovascular outcomes in adult men (Virtanen et al. 2005). In univariate analysis, median hair Hg concentrations were significantly higher among web participants than among in-person participants (1.1 vs. 0.58 μg/g; Wilcoxon rank-sum *p* < 0.0001), marginally higher among men than among women (0.83 vs. 0.71 μg/g; Wilcoxon rank-sum *p* = 0.06), and positively associated with education (0.63 μg/g in the lowest category vs. 1.2 μg/g in the highest; Kruskal-Wallis *p* < 0.0001). Hair Hg was not associated with age, race/ethnicity, or BMI (Table 1).

### Levels and sources of fish consumption and estimated Hg dose

When anglers were asked about their “overall” fish consumption (finfish and shellfish) over the past 3 months, 7% reported eating fish once per month or less, 55% ate fish once per week, 36% ate fish three times per week, and 2% ate fish once per day or more (Table 1). Based on these categorical data, and assuming a standard portion size of 129 g (U.S. EPA 1997), 93% of anglers had an average daily consumption rate ≥ 18 g/day (one meal per week or more), and 38% of anglers had an average daily consumption rate ≥ 55 g/day (three or more meals per week). These estimated rates are slightly higher, although within the ranges, of those reported previously for the U.S. general population and subpopulations of recreational anglers ([Bibr b40-ehp-119-245][Bibr b44-ehp-119-245]).

**Figure 1:**
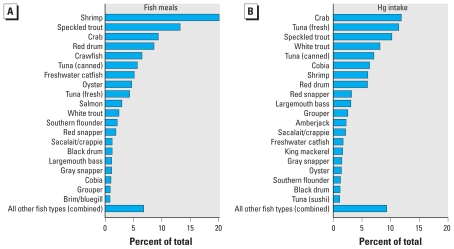
Percent of total fish meals (*A*) and Hg intake (*B*) contributed by individual fish types to total intake across all Louisiana recreational anglers.

However, anglers’ fish consumption patterns differed considerably from the U.S. general population, who primarily eat commercial fish and derive their Hg from these sources (Sunderland 2007). In response to species-specific fish consumption questions, anglers reported consuming 79 types of fish, including finfish and shellfish from recreational and commercial sources. The most commonly consumed fish types were shrimp, speckled trout, crab, red drum, and crawfish. These five types accounted for 57% of the total fish meals consumed by all participants (Figure 1A). Estimated Hg intake was dominated by crab, fresh tuna, speckled trout, white trout, and canned tuna (Figure 1B). These five types accounted for 49% of the total estimated Hg intake by all participants.

Overall, finfish accounted for 62% of meals and 80% of estimated Hg intake, whereas shellfish accounted for 38% of meals and 20% of estimated Hg intake. The lower contribution of shellfish to estimated Hg intake is due to generally lower concentrations of Hg in shellfish [see Supplemental Material, Table 1 (doi:10.1289/ehp.1002609)]. Anglers were queried about the general source of the finfish and shellfish in their diets, whether recreationally caught or commercially bought. Eighty-seven percent of participants reported that all or most of the finfish they consumed was caught recreationally, and 26% reported that all or most of the shellfish they consumed was caught recreationally. When we combined this source information with anglers’ species-specific consumption and dose information, 64% of fish meals and 74% of estimated Hg intake by all participants came from recreational sources [see Supplemental Material, Section 4 (doi:10.1289/ehp.1002609)]. Sixty-five percent of estimated Hg intake derived from recreationally caught finfish, whereas 8% derived from recreationally caught shellfish. The remainder of estimated Hg intake came from commercially bought seafood, with the largest single contribution (7%) coming from canned tuna.

The mean reported number of “species-specific” fish meals was 0.70 meals per day (equivalent to 90.3 g/day), the median was 0.59 meals per day (76.1 g/day), and the range was 0.021–4.9 meals per day. For some anglers (especially those who reported more than three fish meals per day), consumption estimated from species-specific questions appears to be exaggerated, a problem that has also been noted in other studies (e.g., Björnberg et al. 2005; Cavan et al. 1996). However, the species-specific fish consumption questions still offer a valuable qualitative measure of anglers’ diets, providing information on the range of species consumed, the proportion each species contributed to the overall diet, and the source of the fish, whether local or remote [for distributions of total and species-by-species fish consumption, see Supplemental Material, Tables 2 and 3 (doi:10.1289/ehp.1002609)].

We calculated two Hg dose metrics from consumption information and fish Hg concentration data. The “species-specific” Hg dose, which represents dose based purely on reported species-specific fish consumption (Equation 1), had a median of 0.11 μg/kg/day (range, 0.0011–1.2 μg/kg/day). The “scaled” Hg dose, which adjusts species-specific fish consumption relative to overall reported fish consumption (Equation 2), had a median of 0.009 μg/kg/day and a range of 0–0.27 μg/kg/day [for distributions, see Supplemental Material, Table 2 (doi:10.1289/ehp.1002609)].

### Association of hair Hg with fish and estimated Hg intake

We developed multivariate statistical models to evaluate the associations between hair Hg and measures of fish and Hg intake. We considered overall fish consumption, species-specific Hg dose, and scaled Hg dose each as the main effect in separate regression models (Table 2). Each of these metrics was positively and significantly associated with the natural log of hair Hg concentration after controlling for age, sex, race/ethnicity, BMI, education level, and survey type. Moving from the lowest to the highest overall fish consumption category was associated with a 1.1-unit increase in log-transformed hair Hg; a 0.1 μg/kg/day increase in species-specific Hg dose was associated with a 0.18-unit increase in log-transformed hair Hg; and a 0.1 μg/kg/day increase in scaled Hg dose was associated with a 1-unit increase in log-transformed hair Hg. Survey type, sex, and education level were also significantly associated with the natural log of hair Hg in each of the regression models: participants who took the web-based survey, male participants, and participants with higher education levels had higher hair Hg levels than did other participants, even after controlling for the main effect of fish consumption or Hg dose (Table 2). We performed a test for trend for overall fish consumption by assigning an equivalent continuous number of fish meals per month to each category. The trend was significant (*p* = 0.01), and a visual inspection of hair Hg levels by fish consumption category confirms a modest positive association [see Supplemental Material, Figure 1 (doi:10.1289/ehp.1002609)]. Adjusted model *R*^2^ values ranged from 0.17 (overall fish consumption) to 0.25 (species-specific Hg dose).

In addition to the multivariable regressions, we explored the relationship between participants’ estimated Hg doses (both “species-specific” and “scaled”) and their actual hair Hg concentrations in order to assess the accuracy of the FFQ [see Supplemental Material, Section 5 (doi:10.1289/ehp.1002609)]. We entered estimates of anglers’ daily Hg dose into a one-compartment model (U.S. EPA 2001) to calculate predicted hair Hg concentrations, which we then compared with measured hair Hg concentrations [see Supplemental Material, Figure 3 (doi:10.1289/ehp.1002609)]. Although the species-specific Hg dose (Equation 1) and the scaled Hg dose (Equation 2) each predicted hair Hg with comparable efficiency in multivariable regressions (Table 2), they produced very different predicted hair Hg concentrations. For the species-specific dose variable, the slope of the least-squares line fit to the plot of predicted versus measured hair Hg was 13.4 [see Supplemental Material, Figure 3A (doi:10.1289/ehp.1002609)]. This substantial departure from the 1:1 line (where the data should fall if the Hg dose variables perfectly predicted measured hair Hg) further reinforces our observation of overreporting in species-specific questions. By contrast, the scaled Hg dose variable, which we developed to address this potential overreporting, produced a plot with a least-squares line slope of 1.6, much closer to the 1:1 line [see Supplemental Material, Figure 3B (doi:10.1289/ehp.1002609)].

## Discussion

### Hg exposure and fish consumption

Louisiana recreational anglers who participated in this study had a median hair Hg concentration of 0.81 μg/g, approximately four times the median of the only available nationally representative sample of women of childbearing age (0.19 μg/g; McDowell et al. 2004). Anglers’ exposure levels are consistent with a study of high fish-consuming recreational anglers in Montreal (median = 0.82 μg/g; Kosatsky et al. 2000) and with other studies of highly exposed subpopulations in the United States (e.g., household members of fishing license holders in Wisconsin; median = 0.86 μg/g; Knobeloch et al. 2007).

Participants also reported eating fish at somewhat elevated rates, and this consumption level is generally consistent with other studies among residents (including anglers) of southern Louisiana (e.g., Dellenbarger et al. 1993). Participants consumed a wide range of fish types, many of which are regionally specific, and derived most of their Hg from recreationally caught fish. At the national level, estimated Hg intake is driven by a few commonly consumed fish types with moderate to high concentrations of Hg, such as canned tuna (0.35 μg/g) and swordfish (0.98 μg/g) (Sunderland 2007). Conversely, estimated Hg intake among study participants was driven by high consumption of moderate- to low-Hg fish types, including crab (0.18 μg/g), fresh tuna (0.38 μg/g), and speckled trout (0.11 μg/g). The most recent advisory issued by the State of Louisiana for the Gulf of Mexico has recommended limiting consumption of four high-Hg species (king mackerel, cobia, blackfin tuna, and greater amberjack) and is based on an assumed consumption rate of no more than four meals per month (Louisiana Department of Health and Hospitals 2006). However, 38% of study participants reported eating fish more than four times per month (i.e., more than once per week). Furthermore, only 2% of meals and 11% of estimated Hg ingested by participants came from species named in the advisory (Figure 1).

### Differences between survey types

We recruited participants through an Internet survey as well as through in-person interviews to efficiently increase the sample size, recognizing that this approach could result in sampling from two different subgroups of recreational anglers. Comparisons of the two survey groups revealed significant differences in hair Hg (Table 1), education level, and estimated Hg dose [see Supplemental Material, Tables 2, 4 (doi:10.1289/ehp.1002609)], as well as qualitative differences in dietary composition [see Supplemental Material, Figure 2 (doi:10.1289/ehp.1002609)]. In particular, in-person anglers reported higher consumption rates of shellfish (lower in Hg), whereas web-based anglers reported higher consumption rates of finfish (higher in Hg). After controlling for several of these factors in multivariable regressions, the difference in hair Hg between the groups was reduced, although it remained significant [Table 2; for a comparison of adjusted and unadjusted effect estimates, also see Supplemental Material, Section 6 (doi:10.1289/ehp.1002609)]. Other studies have also identified persistent differences in MeHg exposure between demographic subgroups, even after controlling for fish consumption, for example, higher exposures among higher-income compared with lower-income women (Mahaffey et al. 2009) and among Asian (McKelvey et al. 2007) and Inuit (Canuel et al. 2006) fish consumers compared with other ethnic groups. Further description of differences between the survey groups, including stratified results, is included in the Supplementary Material [see Section 6, Tables 2 and 4–6, Figure 3 doi:10.1289/ehp.1002609)]. Overall, our results demonstrate that online recruitment and surveying are valuable tools for studying large populations cost-effectively; however, care must be taken to account for underlying differences between participants surveyed online and those surveyed in person.

### Efficiency and accuracy of exposure predictors

Our results also offer insights into the relative efficiency and accuracy with which MeHg exposure, as measured by a reliable biomarker such as hair Hg, can be explained by dietary recall data. When collection of a biomarker is not feasible, surveys of recent fish consumption are commonly used as a proxy for MeHg exposure (e.g., Mariën and Patrick 2001). Therefore, it is important to understand the relationships between indirect measures, such as surveys of fish consumption or modeled Hg intake based on such surveys, and the relationship of each to Hg biomarkers. This will make studies of MeHg as efficient and accurate as possible.

When anglers were asked about their fish consumption on a species-by-species basis, they reported eating fish with surprising frequency. The highest total consumption was equivalent to more than three meals per day, which suggests some level of overreporting at the high end of the distribution. Other studies have found similar overreporting of species-specific fish consumption (e.g., Björnberg et al. 2005), and nutrition research suggests that overreporting is more common when the number of items on a FFQ is high (Krebs-Smith et al. 1995), as was the case with our species-specific questions. Conversely, underreporting has been found when multiple items are grouped into one category (Serdula et al. 1992), as was the case with our overall consumption question. True fish consumption rates may therefore fall somewhere between the rates reported in the overall question and those reported in the species-specific questions.

Metrics of fish consumption and Hg dose in our study were positively and significantly associated with anglers’ log-transformed hair Hg concentrations when considered in separate multivariable regression models, but each explained only approximately 20% of the variability (*R*^2^ = 0.17–0.25; Table 2). This is not unusual for a biomarker study (e.g., McDowell et al. 2004), but it does suggest that, for MeHg, dietary recall data can be an imprecise measure of exposure. Interestingly, the *R*^2^ values of models containing Hg dose variables were very similar to the *R*^2^ of the model containing overall fish consumption, although the associations were more significant for the former than for the latter (Table 2). This suggests that obtaining a more complex and data-rich exposure metric, such as Hg dose, improves the strength of the exposure–biomarker relationship but may not explain substantially more of the exposure variance than basic dietary data. This may be due to the added imprecision associated with reported intakes of multiple species or to the use of point estimates for fish Hg concentrations, which are known to vary considerably with fish size and age.

Finally, a comparison of anglers’ measured hair Hg concentrations with those predicted by their estimated species-specific and scaled Hg doses suggested that the scaled Hg dose variable best approximates the measured exposure and may correct some of the potential overreporting in the species-specific fish consumption variable. This finding suggests that FFQs by themselves may not be sufficient to assess MeHg exposure and that calibration or validation of the exposure metric with a biomarker such as hair Hg is necessary.

## Conclusions

Study participants had high levels of MeHg exposure. Their exposure was dominated by a wide range of recreationally caught fish, many of which are regionally specific. In contrast, MeHg exposure for the average U.S. fish consumer is driven by consumption of a few commercial, widely available fish species (Sunderland 2007). National advisories crafted to reduce MeHg exposure, such as the recent joint EPA/FDA fish consumption recommendation (U.S. EPA 2004), are not designed to protect populations whose exposure pathways are unique or regionally specific. Even locally issued fish consumption advisories that target high-Hg species may not be adequate, although if followed they are likely to reduce exposures in these populations. Among this study’s participants, results suggest that exposure was driven not by high-Hg species but by consumption of low- to moderate-Hg species. More work is needed to characterize other highly MeHg-exposed subpopulations in the United States and elsewhere.

Our study design resulted in successful recruitment both through in-person interviews and through an online survey. Internet-based surveys can be an efficient way to recruit larger numbers of participants, and our results demonstrate that these participants can reliably provide biomarker samples. However, care must be taken to account for any underlying differences between study participants surveyed in person and those surveyed online.

Finally, in addition to specific findings for Louisiana recreational anglers, our research sheds light on the utility of and relationships among various measures of MeHg exposure. Our findings suggest that FFQ data alone may not be sufficient to quantify MeHg exposure, and that hair samples—which are easily collected alongside a survey and remain the standard for MeHg exposure assessment—provide a better measure of overall exposure. In a uniquely exposed population, such as the one characterized here, the best approach of all may be one that combines biomarker data with species-specific FFQ data, allowing for characterization of the magnitude as well as the sources of the exposure.

## Figures and Tables

**Table 1:** Louisiana recreational anglers’ hair Hg concentrations stratified by demographic variables and quartiles of fish consumption and estimated Hg dose.

					Percentile		
Angler group	*n*	Mean ± SD	Range	Median	75th	90th	95th
All	398	1.1 ± 1.1	0.02–10.7	0.81	1.4	2.3	3.2

Sex							
Male	354	1.2 ± 1.1	0.02–10.7	0.83	1.5	2.3	3.2
Female	44	0.91 ± 0.81	0.05–3.6	0.71	1.1	1.9	2.5

Age (years)							
18–39	106	1.3 ± 1.1	0.02–5.0	0.98	1.8	2.6	3.9
40–54	176	1.1 ± 1.2	0.05–10.7	0.80	1.3	2.2	3.3
55–84	114	0.97 ± 0.76	0.08–4.8	0.73	1.2	1.8	2.5

Race/ethnicity							
White	381	1.1 ± 1.1	0.02–10.7	0.81	1.4	2.3	3.2
Nonwhite	17	1.2 ± 1.2	0.05–4.9	0.74	1.6	2.4	3.0

Education							
≤ High school degree	106	0.82 ± 0.83	0.02–6.6	0.63	1.0	1.5	2.0
Some college^a^	88	0.93 ± 0.63	0.13–3.4	0.77	1.2	1.8	2.2
College degree	122	1.3 ± 1.3	0.09–10.7	0.87	1.7	2.5	3.6
Postcollege or graduate	82	1.6 ± 1.2	0.08–6.8	1.2	2.0	3.4	4.0

Survey type							
In person	177	0.73 ± 0.49	0.02–2.4	0.58	0.96	1.5	1.8
Web	221	1.5 ± 1.3	0.08–10.7	1.1	1.9	3.1	4.0

BMI (kg/m^2^)							
< 25	102	1.1 ± 0.93	0.05–4.6	0.83	1.5	2.3	3.2
25–29.9	184	1.1 ± 0.89	0.13–5.0	0.87	1.4	2.2	2.7
≥ 30	111	1.2 ± 1.5	0.02–10.7	0.73	1.3	2.4	3.7

Fish consumption							
≤ 1×/month or less	23	0.93 ± 0.80	0.08–3.7	0.75	1.2	1.8	2.2
1×/week	211	1.1 ± 1.0	0.09–6.6	0.78	1.4	2.4	3.4
3×/week	158	1.2 ± 1.2	0.02–10.7	0.88	1.5	2.1	2.4
≥ 1/day	6	2.3 ± 1.5	0.65–4.9	1.8	2.9	4.0	4.5

Species-specific Hg dose (μg/kg/day)							
Q1 (0.0011–0.062)	95	0.68 ± 0.76	0.02–6.6	0.52	0.75	1.2	1.6
Q2 (0.062–0.11)	103	1.0 ± 0.86	0.12–4.8	0.73	1.2	2.1	2.8
Q3 (0.11–0.18)	102	1.2 ± 0.96	0.08–6.8	0.99	1.6	2.1	2.9
Q4 (0.18–1.18)	97	1.7 ± 1.4	0.05–10.7	1.3	2.1	3.3	4.5

Scaled Hg dose (μg/kg/day)							
Q1 (0–0.0052)	96	0.69 ± 0.55	0.08–3.7	0.55	0.82	1.2	1.6
Q2 (0.0052–0.0091)	95	1.3 ± 1.1	0.09–6.6	0.91	1.6	2.5	3.6
Q3 (0.0091–0.023)	100	1.1 ± 97	0.02–5.0	0.84	1.4	2.3	3.2
Q4 (0.023–0.27)	106	1.5 ± 1.4	0.05–10.7	1.2	1.8	2.5	3.6

Q1–Q4 indicate first through fourth quartile. Age data were missing for two participants, and BMI and scaled Hg dose data were missing for one participant.

a Includes vocational/technical school and associate’s degree.

**Table 2:** Linear regression of the natural log of Louisiana recreational anglers’ hair Hg concentration on three fish consumption and Hg dose metrics (each metric modeled separately).

Model/variable	β-Coefficient	95% confidence interval	*p*-Value	Adjusted model *R*^2^
Overall fish consumption				0.17

Frequency				
≤ 1×/month	Referent	—	—	
1×/week	0.32	−0.014–0.65	0.061	
3×/week	0.38	0.037–0.71	0.030*	
≥ 1×/day	1.1	0.37–1.8	0.003*	
Age (years)	−0.0024	−0.0084–0.0036	0.438	
BMI (kg/m^2^)	−0.0047	−0.021–0.011	0.562	
Survey type				
In person	Referent	—	—	
Web	0.47	0.31–0.64	< 0.001*	
Sex				
Female	Referent	—	—	
Male	0.26	0.0093–0.51	0.042*	
Race/ethnicity				
White/Caucasian	Referent	—	—	
Nonwhite	−0.012	−0.39–0.37	0.948	
Education level				
≤ High school degree	Referent	—	—	
Some college	0.074	−0.15–0.30	0.517	
College degree	0.23	0.011–0.44	0.040*	
Postgraduate	0.45	0.21–0.70	< 0.001*	

Species-specific Hg dose				0.25

Daily dose (μg/kg/day)	1.8	1.3–2.4	< 0.001*	
Age (years)	0.00039	−0.0054–0.0061	0.893	
BMI (kg/m^2^)	0.0040	−0.011–0.019	0.608	
Survey type				
In person	Referent	—	—	
Web	0.40	0.24–0.56	< 0.001*	
Sex				
Female	Referent	—	—	
Male	0.36	0.12–0.60	0.003*	
Race/ethnicity				
White/Caucasian	Referent	—	—	
Nonwhite	0.085	−0.28–0.45	0.645	
Education level				
≤ High school degree	Referent	—	—	
Some college	0.099	−0.11–0.31	0.361	
College degree	0.22	0.020–0.43	0.031*	
Postgraduate	0.42	0.19–0.66	< 0.001*	

Scaled Hg dose				0.21

Daily dose (μg/kg/day)	10	6.3–14	< 0.001*	
Age (years)	−0.0016	−0.0075–0.0042	0.585	
BMI (kg/m^2^)	0.0011	−0.014–0.017	0.887	
Survey type				
In person	Referent	—	—	
Web	0.46	0.29–0.62	< 0.001*	
Sex				
Female	Referent	—	—	
Male	0.32	0.078–0.56	0.010*	
Race/ethnicity				
White	Referent	—	—	
Nonwhite	0.089	−0.28–0.46	0.635	
Education level				
≤ High school degree	Referent	—	—	
Some college	0.084	−0.13–0.30	0.448	
College degree	0.20	−0.13–0.40	0.066	
Postgraduate	0.42	0.18–0.66	< 0.001*	

* *p* < 0.05.
